# What Happens If Hepatic Veins Drain to the Pulmonary Venous Atrium? Depends on the Physiology!

**DOI:** 10.1016/j.jaccas.2025.103305

**Published:** 2025-03-12

**Authors:** Kali A. Hopkins, Barry A. Love

**Affiliations:** a Department of Medicine, Icahn School of Medicine at Mount Sinai, Mount Sinai Beth Israel, New York, New York, USA; b Mount Sinai Heart, Icahn School of Medicine at Mount Sinai, New York, New York, USA

**Keywords:** atrial septal defect, congenital heart defect, occlude

## Abstract

In rare instances in congenital cardiac surgery, ≥1 of the hepatic veins may end up incorporated either intentionally or inadvertently with the pulmonary venous atrium (oxygenated blood) while the remainder of the hepatic veins are left to drain normally with the systemic venous blood (less oxygenated blood). What happens when this is done? As it turns out, hepatic venous connections end up forming between the lobes of the liver, and a significant shunt results. The direction of shunting, however, depends on the physiology. We present 2 cases in which a single hepatic vein was left connected to the pulmonary venous atrium and produced very different hemodynamic consequences.

## Graphical Abstract

**Figure:**
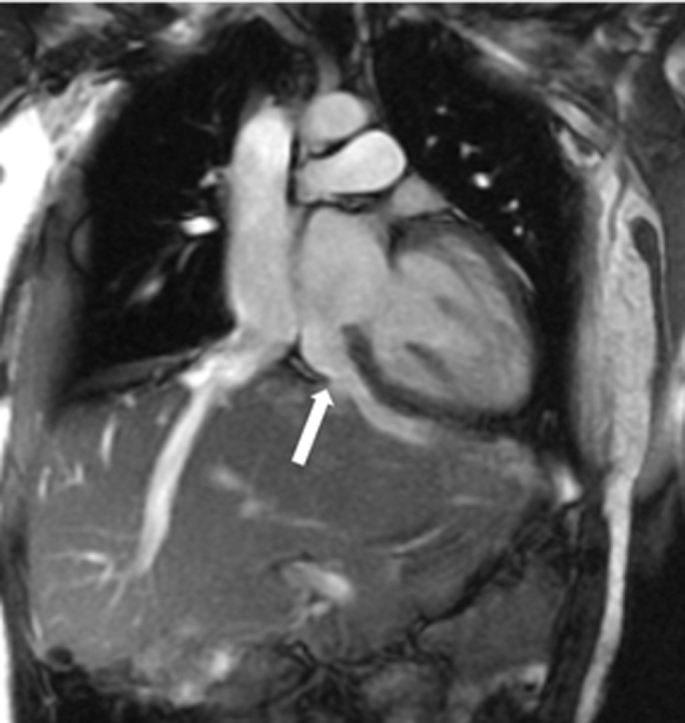

## Case Presentations

### Patient 1

A 23-year-old woman with a history of an atrioventricular septal defect and heterotaxy syndrome underwent pulmonary artery banding in infancy followed by debanding and complete repair at age 2 years. Her systemic venous anatomy consisted of an interrupted inferior vena cava (IVC) with azygous vein continuation to the right-sided superior vena cava (SVC), SVC and right hepatic veins connected to the right-sided atrium, and the left hepatic vein (LHV) connected to the left atrium (LA). During septation of the atrium during the surgical repair, it was decided to leave the left hepatic vein draining to the left atrium because it was difficult to incorporate on the systemic side and was not expected to cause problems.

**Take-Home Messages**

• Significant shunting can develop through enlarging hepatic channels when a hepatic vein is left draining to the pulmonary venous atrium.
• The direction of blood flow can be left-to-right or right-to-left, depending on the physiology.
• Closure of the hepatocardiac connection may be feasible with a transcatheter approach and resolves the shunt.

**Figure 1:**
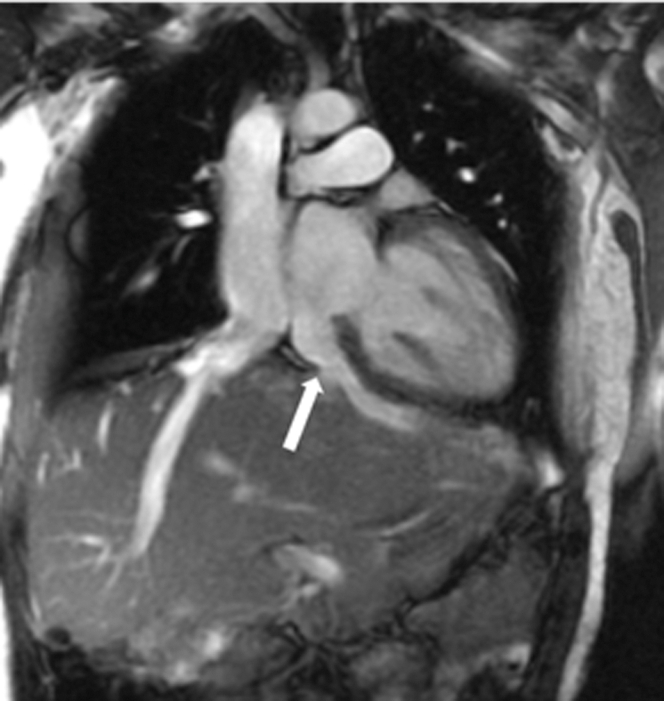
Anomalous Left Hepatic Vein to Left Atrium Magnetic resonance imaging showing a midline liver with left hepatic vein draining directly to the left atrium (arrow) and a right hepatic vein connecting to the suprahepatic inferior vena cava that eventually drains to the right atrium.

**Figure 2:**
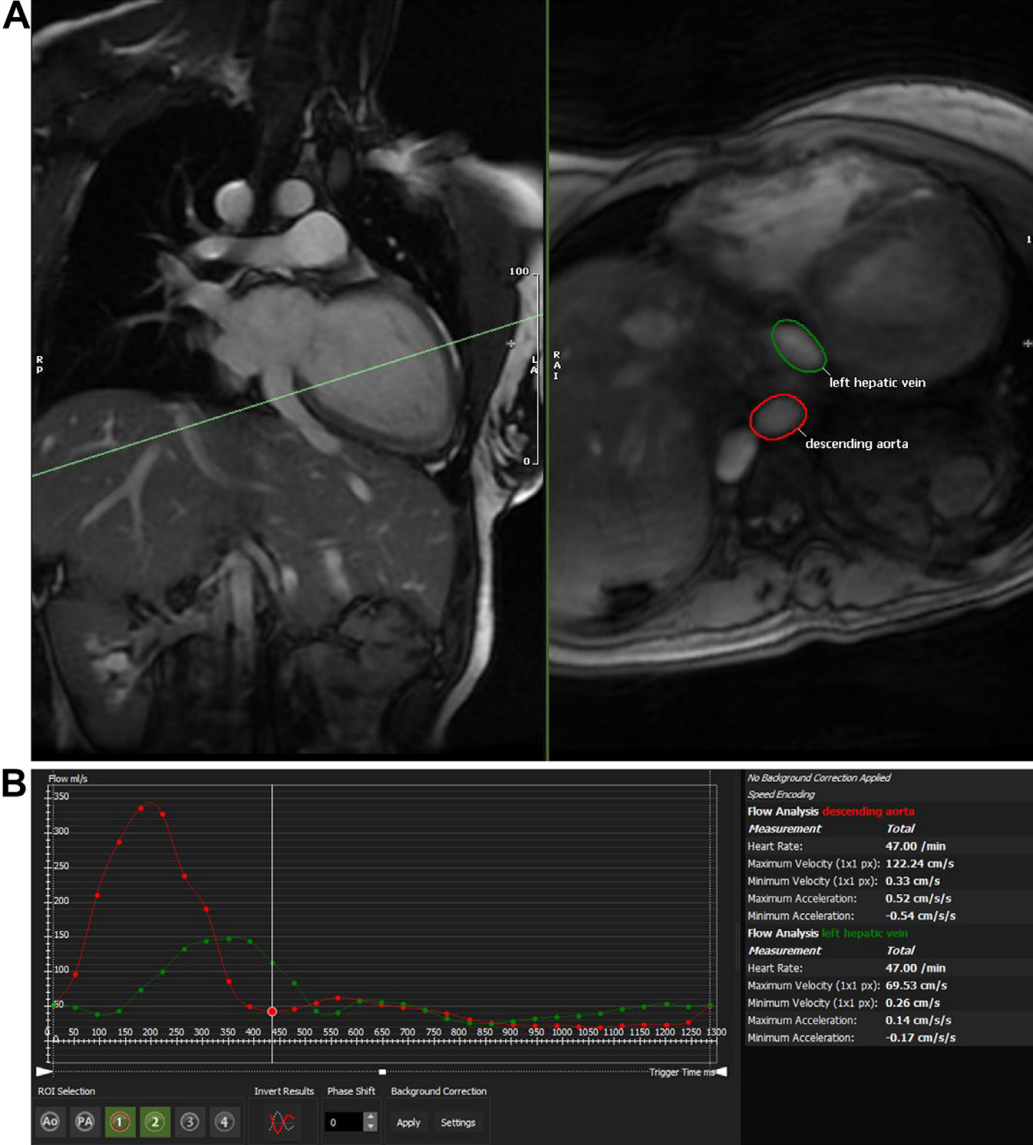
Anomalous Left Hepatic Vein Flow Data (A) Magnetic resonance phase-contrast imaging shows the oblique plane at which the data were obtained, which was perpendicular to the anomalous left hepatic vein connected to the left atrium. The green circle shows where the left hepatic venous flow was sampled in cross-section; the red circle shows the same for the descending aorta. (B) Flow analysis from the phase-contrast imaging confirms direction of flow from the left hepatic vein toward the liver (green), the same direction as the descending aorta (red).

She was asymptomatic but had progressive right heart dilatation on routine follow-up. Cardiac magnetic resonance (CMR) showed a Qp:Qs of 1.4:1 based on flow comparison of the pulmonary artery to the aorta and a dilatated right ventricle (122 mL/m^2^). The LA and left ventricle measured normal in size. There were dilatated hepatic sinusoids connected to the anomalous LHV (Figure 1). No additional shunt, such as residual atrial communication or partial anomalous pulmonary venous return, was identified. Phase contrast magnetic resonance images perpendicular to the anomalous LHV (Figure 2) confirmed flow from the left atrium to the liver.

Cardiac catheterization (Video 1A) confirmed blood flow from the LA to the LHV through the sinusoids to the right hepatic venous system by angiography. Direct pressure measurements supported this diagnosis by demonstrating a mean left atrial pressure of 12 mm Hg, which was greater than the right ventricular end-diastolic pressure of 10 mm Hg. The Qp:Qs was consistent with CMR at 1.5:1 (based on oxygen saturations of 71% in the SVC, 81% in the pulmonary artery, 95% in the descending aorta, and 97% in the left atrium).

The entrance of the LHV to the LA was occluded with a 28-mm Amplatzer septal occluder (Abbott) as shown in Video 1B. After closure of the hepatic vein, the Qp:Qs normalized to 1:1 (79% in the SVC, 81% in the pulmonary artery, presumed 97% in the left atrium, and 95% in the descending aorta). The right ventricular size improved down to 106 mL/m^2^ on follow-up CMR. There were no signs of hepatic congestion on follow-up.

### Expert commentary

Heterotaxy syndrome is a rare congenital syndrome occurring in approximately 1 in 10,000 live births. The term comprises a constellation of abnormalities involving the lateralization of abdominal and thoracic organs across the left-to-right axis.^1^^,^^2^ Interruption of the IVC with azygous continuation to the SVC typically occurs in patients with left atrial isomerism with or without an atrial septal defect. This patient had essentially a common atrium before repair. Hepatic veins can connect to 1 or both sides of the common atrium,^1^ as seen in this patient. In this scenario, 1 hepatic vein connected to the LA and the others drained appropriately to the RA. The hepatic veins are connected by a network of small sinusoids. Similar to the physiology of an atrial septal defect, shunting is determined by the difference in ventricular compliance. In this case, the left ventricular end-diastolic pressure was equal to the LA pressure of 12 mm Hg, which was greater than the right ventricular end-diastolic pressure of 10 mm Hg. Because of this pressure difference, a left-to-right shunt was created, and the high volume through the hepatic sinusoids caused them to dilate. After complete occlusion, the ventricular size normalized as a result of elimination of the shunt, and the LHV drained to the right hepatic vein via the existing sinusoids, thereby avoiding liver congestion.

### Patient 2

An 11-year-old boy had been born with hypoplastic left heart syndrome and had undergone single-ventricle palliation, first with a Norwood procedure (anastomosis of the main pulmonary artery to the aorta and placement of a Blalock-Thomas-Taussig shunt) followed by a bilateral Glenn procedure (anastomosis of the SVC to the right pulmonary artery and takedown of the shunt). At age 4 years, he underwent the final surgical staged procedure (lateral tunnel Fontan) whereby the IVC blood is baffled through the right atrium to the undersurface of the pulmonary artery. At that surgery, in lieu of a “fenestration” in the baffle, the decision was made to intentionally not incorporate the LHV in the Fontan circulation that was directed to the pulmonary artery but rather to leave it on the pulmonary venous side of the baffle as an intentional small right-to-∖left shunt. By age 11 years, his arterial oxygen saturation had decreased significantly to 88%, and he was symptomatic with exercise intolerance.

Cardiac catheterization with angiography of the Fontan circulation showed blood flowing from the Fontan through dilated hepatic sinusoids to the pulmonary venous atrium, creating a right-to-left shunt (Video 2). The hemodynamics supported this diagnosis with a mean Fontan/IVC pressure of 9 mm Hg compared with a pulmonary venous atrial pressure of 6 mm Hg. The orifice of the left hepatic vein to the pulmonary venous atrium was closed with multiple coils (Videos 3A and 3B), improving the oxygen saturation to 98%.

### Expert commentary

In the early 1990s, some surgeons advocated for partial hepatic venous exclusion from the Fontan circulation in an effort to reduce postoperative pleural and pericardial effusions in lieu of a surgically created fenestration.^3^ Early postoperative outcomes were favorable at reducing the frequency and severity of effusion.^4^ However, there have been several reports of increasing cyanosis caused by increasing right-to-left shunting^4^, ^5^, ^6^ from the chronic pressure gradient from the Fontan to the atrium, resulting in dilatated hepatic channels, as was seen in this patient. Although limited to case reports and case series, the patients described in the literature who experienced this form of intrahepatic steal underwent surgical re-intervention to baffle the hepatic vein to the IVC.^4^, ^5^, ^6^, ^7^ There were later reports of transcatheter hepatocardiac venous occlusion after the Fontan procedure.^7^, ^8^, ^9^, ^10^, ^11^, ^12^, ^13^ In addition to these cases, our case demonstrates successful occlusion of the hepatic vein with improved oxygen saturation and no adverse consequences for venous drainage of the liver.

## Discussion

Surgical exclusion of the left hepatic vein to the pulmonary venous atrium leads to enlarging hepatic venous channels because of the difference in systemic and pulmonary venous pressures. Both cases demonstrated progressive dilatation of the intrahepatic venous channels and thus equalization of pressure within the different hepatic lobes. The direction of shunt (left-to-right or right-to-left) depends on the downstream physiology. The first patient showed left-to-right shunting caused by the higher left ventricular end-diastolic pressure compared with the right ventricular end-diastolic pressure, akin to what one would expect in atrial septal defect shunts. Patient 2 showed right-to-left shunting because the pressure in the Fontan circulation is obligatorily higher than the left atrial pressure. Occlusion of the hepatocardiac connection resolves the shunt without causing hepatic congestion because the occluded hepatic vein is able to decompress through the existing hepatic sinusoidal network.

## Funding Support and Author Disclosures

The authors have reported that they have no relationships relevant to the contents of this paper to disclose.

## Appendix

Video 1a (A) Angiogram with left atrial injection showing reversal of blood flow from the left atrium to the left hepatic vein that drains via dilated sinusoids to the right hepatic vein to the right atrium. (B) Following deployment of the Amplatzer Septal Occluder device, injection in the left hepatic vein shows no residual connection from the left hepatic vein to the left atrium and unobstructed flow from the left hepatic vein to the right hepatic vein via dilated sinusoids.

Video 1b (A) Angiogram with left atrial injection showing reversal of blood flow from the left atrium to the left hepatic vein that drains via dilated sinusoids to the right hepatic vein to the right atrium. (B) Following deployment of the Amplatzer Septal Occluder device, injection in the left hepatic vein shows no residual connection from the left hepatic vein to the left atrium and unobstructed flow from the left hepatic vein to the right hepatic vein via dilated sinusoids.

Video 2a Angiogram via balloon wedge catheter in the Fontan baffle shows venous collateral channels through the liver to the left hepatic vein that drains to the common atrium shown in the anterior-posterior (A) and lateral projections) creating a right-to-left shunt. The pigtail catheter is positioned in the descending aorta.3

Video 2b Angiogram via balloon wedge catheter in the Fontan baffle shows venous collateral channels through the liver to the left hepatic vein that drains to the common atrium shown in the anterior-posterior (A) and lateral projections) creating a right-to-left shunt. The pigtail catheter is positioned in the descending aorta.

Video 3A (A) Direct left hepatic vein angiogram shows drainage from the vein to the right atrium.

Video 3B Left hepatic vein angiogram after coil occlusion shows no residual flow into the atrium and unobstructed flow to the Fontan circulation.
